# A Novel Enterovirus 71 (EV71) Virulence Determinant: The 69th Residue of 3C Protease Modulates Pathogenicity

**DOI:** 10.3389/fcimb.2017.00026

**Published:** 2017-02-03

**Authors:** Bingqing Li, Yingying Yue, Yajie Zhang, Zenglin Yuan, Peng Li, Nannan Song, Wei Lin, Yan Liu, Lichuan Gu, Hong Meng

**Affiliations:** ^1^Key Laboratory of Rare and Uncommon Diseases, Department of Microbiology, Institute of Basic Medicine, Shandong Academy of Medical SciencesJinan, China; ^2^State Key Laboratory of Microbial Technology, School of Life Sciences, Shandong UniversityJinan, China

**Keywords:** human enterovirus type 71, 3C protease, N69D substitution, crystal structure, virulence determinantion

## Abstract

Human enterovirus type 71 (EV71), the major causative agent of hand-foot-and-mouth disease, has been known to cause fatal neurological complications. Unfortunately, the reason for neurological complications that have been seen in fatal cases of the disease and the relationship between EV71 virulence and viral genetic sequences remains largely undefined. The 3C protease (3C^pro^) of EV71 plays an irreplaceable role in segmenting the precursor polyprotein during viral replication, and intervening with host life activity during viral infection. In this study, for the first time, the 69th residue of 3C protease has been identified as a novel virulence determinant of EV71. The recombinant virus with single point variation, in the 69th of 3C^pro^, exhibited obvious decline in replication, and virulence. We further determined the crystal structure of 3C N69D at 1.39 Ǻ resolution and found that conformation of 3C N69D demonstrated significant changes compared with a normal 3C protein, in the substrate-binding site and catalytic active site. Strikingly, one of the switch loops, essential in fixing substrates, adopts an open conformation in the 3C N69D-rupintrivir complex. Consistent with this apparent structural disruption, the catalytic activity of 3C N69D decreased sharply for host derived and viral derived substrates, detected for both *in vitro* and *in vivo*. Interestingly, in addition to EV71, Asp69 was also found in 3C proteases of other virus strains, such as CAV16, and was conserved in nearly all C type human rhinovirus. Overall, we identified a natural virulence determinant of 3C protease and revealed the mechanism of attenuated virulence is mediated by N69D substitution. Our data provides new insight into the enzymatic mechanism of a subdued 3C protease and suggests a theoretical basis for virulence determinantion of picornaviridae.

## Introduction

Enterovirus 71 (EV71) is the major causative agent of hand-foot-and-mouth disease (HFMD) particularly aiming young children and infants (Shindarov et al., 1979; Lum et al., 1998; Ho, 2000; Yang et al., 2009). This virus belongs to the picornaviridae family and consists of a ~7400 bp positive single-stranded RNA and exhibits frequent variation (Lum et al., 1998; Ho, 2000). Since the first reported HFMD outbreak in California in 1969, it has become a continuing threat to global public health especially in the Asia-Pacific region. After the unusual HFMD outbreak in 2008, HFMD was designated one of the national infectious diseases of China due to its substantial morbidity and mortality (Yang et al., 2009). The total number of HFMD was reported of about 12,800,000 cases by the end of 2015 in China mainland, including about 580,000 severe cases and 3296 fatal cases. The clinical characterizations of HFMD are prodromal fever followed by pharyngitis, mouth herpes, and rash on the hands and feet (Huang et al., 1999; Ho, 2000). Most patients recovered within a week, but a few severe cases suffered from neurological complications including convulsion, ataxia, brain-stem encephalitis, poliomyelitis-like paralysis, and even death (Lum et al., 1998; Huang et al., 1999). Although an inactivated enterovirus 71 vaccine has been developed recently, the efficacy, and safety of this vaccine remain to be further tested (Li et al., 2014; Zhu et al., 2014). The pathogenic mechanism of EV71 remains unclear. Most importantly, the reason for neurological complications of severe cases or fatal cases remains largely undefined. It has been reported that both the host's immune state and viral genetic sequences contribute to the severity of HFMD (Huang et al., 1999; Weng et al., 2010).

In the past, there were a few reports demonstrating that the pathogenicity of EV71 can be enhanced or weakened by a single mutation of its genome. For instance, researchers found that mutation of the receptor interacting region of EV71 VP1 or the palm domain and finger domain of EV71 3D will lead to attenuated virus virulence in the EV71 B or C1 genotype (Meng and Kwang, 2014; Zhang et al., 2014; Yuan et al., 2015). However, for the EV71 C4 genotype, the most predominant EV71 strain currently in China, there is little known about the virulence determinants or the molecular mechanisms underlying the attenuated phenotype. Previously, our group isolated six EV71 strains from patients of Jinan, China during 2008 and 2010 (Li et al., 2016). Those EV71 strains, which were identified as the C4 genotype, were classified into two classes according to the infection-induced fatality of 1-day-old mice. Sequence analysis demonstrated eight substitutions in amino acids between lethal-strains and a non-lethal strain. In this study, we attempted to elucidate the virulence determinants of the EV71 C4 genotype and the molecular basis of the underlying pathogenic difference. To do this, we used a reverse genetics approach to establish eight recombinant viruses with a single amino acid substitution and compared the replication ability of these viruses with wild-type. Interestingly, only the variant with a mutant in the 69th residue of 3C^pro^ exhibited significant differences from the WT strain.

The genome of EV71 encodes a precursor polyprotein of ~2193aa in length which is incised into four viral structural proteins (VP1, VP2, VP3, VP4) and seven functional proteins (2A, 2B, 2C, 3A, 3B, 3C, 3D) during virus replication (Ho, 2000). All the proteolytic processes of EV71 polyprotein are performed by its 3C protease except the cleavage of VP1-2A and VP2-VP4 (Parks et al., 1989; Dasmahapatra et al., 1992). More importantly, like other picornaviral 3C proteases, EV71 3C^pro^ can impair host cellular functions by hydrolysis of host targets (Kuyumcu-Martinez et al., 2004a,b; Strong and Belsham, 2004; Kundu et al., 2005; Bonderoff et al., 2008; De Breyne et al., 2008; Xiang et al., 2014). Wang et al. reported that EV71 3C^pro^ interferes with host RNA polyadenylation by digesting the host protein CstF-64, a cleavage stimulating factor responsible for 3′ pre-mRNA cleavage and polyadenylation (Weng et al., 2009). More recently, EV71 3C^pro^ was found to inhibit host immune responses by cleavage of the adaptor protein TRIF and interferon regulatory factor 7 (IRF7) (Lei et al., 2013). Those findings suggest that EV71 3C^pro^ plays a significant role in both virus replication and virus-host interaction. Our results indicated that mutation of the 69th residue of EV71 3C^pro^ provides a distinct reduction of viral replication to attenuate virulence of the virus. However, the mechanism by which the 3C^pro^ N69D mutant alters virulence has not been determined. For this purpose, we determined the crystal structure of 3C^pro^ N69D at 1.4 Å resolution. Structural analysis combined with enzymatic activity assays revealed dramatic structural and functional changes of 3C^pro^ N69D compared with native 3C^pro^ which may explain the molecular mechanism of the reduced virulence of the EV71 non-lethal strain.

## Materials and methods

### Construction of single mutant EV71 plasmids and transfection

Eight mutational plasmids were amplified from A12 plasmids (infectious full-length cDNA of EV71 containing a SP6 promoter, gifted by the Academy of Military Medical Sciences, Beijing, China) using a Site-Directed Mutagenesis system (TransGen biotech, Beijing, China; Cao et al., 2013). PCR products were digested with DMT enzyme (TransGen) at 37°C for 1 h and transformed into DMT cells (TransGen). Then, Plasmids were digested with MluI Enzyme (Thermo Scientific, Rockford, IL, USA) for 30 min at 37°C and further purified using DNA Purification Kit (Thermo). Linearized DNA was transcribed into RNA using SP6 *in vitro* Transcription Kit (Thermo) according to the instructions. The reaction mixtures were incubated at 37°C for 2 h, followed by treatment with DpnI enzyme (Thermo) at 37°C for 5 min. The transcripts were purified using an RNA Purification Kit (Thermo). About 1 ng RNA transcripts were mixed with moderate Lipofectamine 2000 reagent (Invitrogen, USA) for 15 min at room temperature. The mixtures were then added to Rhabdomyosarcoma (RD) cells or 293T cells in a 24-plate and incubated at 37°C with 5% CO_2_. Cell cultures were harvested when the cytopathic effect (CPE) reached 70–80% and stored at −80°C.

### RT-PCR

Total RNA was extracted from cell cultures infected by rescued virus using the viral RNA Kit (TransGen). The reverse transcription reactions were performed using Revert Aid cDNA Synthesis Kit (Thermo) with Oligo (dT) as primer and incubated at 42°C for 1 h, followed by 10 min at 70°C. RT-PCR was performed by EV71 specific primers (forward primer: 5′-GCAGCCCAAAAGAACTTCAC-3′, reverse primer: 5′-ATTTCAGCAGCTTGGAGTGC-3′) in a volume of 25 μl. After electrophoresis, PCR products were purified using DNA Purification Kit (Thermo) and sequenced (Biosune, Shanghai, China).

### Replication kinetics

RD cells seeded in a 24-well plate with 90% confluency were infected with rescued virus at a Multiplicity of Infection (MOI) of 1 and cultured at 37°C for 1 h, followed by washing three times with PBS to remove unattached virus. Then, 2% FBS-1640 medium was added. Cells and supernatants were sampled at 6, 12, 24, 48, and 60 h post infection. The quantitative RT-PCR reactions were performed on an Applied Biosystems 7500 Sequence Detection system (Applied Biosystems, Foster, CA, USA) to quantify viral RNA copies (Li et al., 2015). The reaction system contained 0.1 μl of cDNA of samples and 12.5 μl of SYBR Green qPCR Master Mix (Thermo), 0.05 μl of 10-fold dilutions of ROX, and 0.3 μM of each EV71 specific primer in 25 μl of reaction volume. The A12 plasmids were serially diluted 10-fold and were used as a reference standard to generate the standard curve and quantify viral RNA copies. All samples were detected in triplicate.

### Western blot and antibodies

RD cells or 293T cells infected with RNA transcripts or plasmids were cultured at 37°C with 5% CO_2_ for different amounts of time and then lysed in a SDS–PAGE loading dye, followed by heating at 95°C for 10 min before separation by SDS-PAGE. Cells infected with wild type EV71 were used as positive controls and uninfected cells were used as negative controls. Total proteins on gels were transferred to nitrocellulose membranes at 250 mA for 2 h in transfer buffer (96 mM glycine, 12.5 mM Tris and 10% methanol). The membranes were blocked in 5% milk soluted in PBS-0.1% Tween 20 (PBST) at 37°C for 1 h, followed by incubation with indicated primary antibodies in PBST over night at 4°C. The membranes were washed three times in PBST and then incubated at 37°C for 1 h in secondary antibody diluted 1:10000 in PBST. The membranes were washed three times for 10 min and then detected by chemiluminescence.

Antibodies against IRF7, NF-κB, HRP Conjugated Goat anti Rabbit IgG H&L and Goat Anti-Mouse IgG H&L were purchased from abcam (UK). Anti-GADPH was purchased from Cell Signaling Technology (Danvers, MA). Anti-EV71 3C and anti-EV71 3D antibodies were purchased from GeneTex (UK).

### Indirect fluorescent-antibody (IFA) assay

RD cells seeded on a slide were infected with rescued virus and cultured for 12 h at 37°C with 5% CO_2_. Cells infected with wild type EV71 were used as positive controls and uninfected cells were used as negative controls. Cells were washed with PBS and fixed in 4% paraformaldehyde (soluted in PBS) at 4°C for 30 min, then blocked in 5% blocking milk for 1 h at room temperature. Then, cells were washed with PBS and incubated over night at 4°C with mouse polyclonal antibody to EV71 (GeneTex) diluted 1:200 in PBS. Cells on slides were washed again before incubation with Goat Anti-Mouse IgG H&L (abcam) diluted 1:500 in PBS at 37°C for 1 h. Finally, RD cells were washed with PBS three times for 5 min and then visualized under a fluorescent microscope.

### Protein expression and purification

The native 3C and 3C N69D genes were amplified from the cDNA of EV71 lethal-strains and non-lethal strain and cloned into the pGL01 vector, a modified vector based on pET15b with a Prescission Protease (PPase) cleavage site to remove the 6 × His tag after purification (Li et al., 2012). The 3C C147S mutant was constructed using a two-step PCR strategy and also cloned into pGL01. The protein-encoding plasmids were grown and expressed in *E. coli* BL21 (DE3) in LB medium with 100 μg/ml Ampicillin. When the OD600 reached 0.6, cultures were cooled to 16°C and induced overnight by addition of 0.1 mM IPTG. Harvested cells were resuspended in lysis buffer (25 mM Tris-HCl pH 8.0, 200 mM NaCl) and lysed by sonication. After centrifugation at 28,500 × g for 45 min, proteins were purified by Ni^2+^-NTA affinity column and the His-tag was removed using PPase for 5 h. The target proteins lacking the His-tag were concentrated to 2 ml and purified by Superdex 200 in buffer containing 10 mM Tris-HCl pH 8.0 and 100 mM NaCl. Purified proteins were stored at −80°C. Genes of the native 3C, 3C N69D, and 3C C147S were also cloned into pFLAG-CMV2 vector for *in vivo* experiment. Recombinant plasmids were transfected into 293 T cells using liposomes. The intracellular expression of IRF7 was detected by immunoblotting.

### Crystallization and structure determination

3C N69D were concentrated to about 10 mg/ml and rupintrivir dissolved in DMSO was added to a final concentration of 3 mM. After incubation on ice for 10 min, the mixtures were centrifuged before crystallization (Wang et al., 2011). The crystals were obtained in the presence of 30% PEG 8000 and 0.1 M sodium acetate (pH 4.5) after 7 days. To prevent radiation damage, crystals were equilibrated in a cryoprotectant buffer containing 15% glycerol (v/v) plus reservoir buffer and then flash frozen in a 100 K nitrogen stream. Diffraction data were collected at Shanghai Synchrotron Radiation facility (SSRF) beamline BL17 ul. The data set was processed using the HKL2000 software suite. The structure was solved by molecular replacement using PHASER with native 3C (PDB: 3SJO) as the searching model. The atomic model was built using Coot and refined using PHENIX (Adams et al., 2002; Emsley and Cowtan, 2004). Data collection and structure refinement statistics are summarized in Table 1. Structural figures were generated using PyMol (http://www.pymol.org).

**Table 1:** **Data collection and refinement statistics**.

	**3C N69D–Rupintrivir**
Resolution range (Ã…)	20.53–1.39 (1.44–1.39)
Space group	*P*2_1_
Unit cell	36.2 64.8 38.3 90 113.1 90
Unique reflections	32549 (3195)
Completeness (%)	99.3 (98.6)
Mean I/sigma (I)	22.38 (4.84)
Wilson B-factor	13.7
R-factor	0.174 (0.228)
R-free	0.199 (0.252)
Number of atoms	1764
macromolecules	1475
ligands	53
water	239
Protein residues	182
RMS(bonds)	0.008
RMS(angles)	1.19
Ramachandran favored (%)	98
Ramachandran outliers (%)	0
Clashscore	4.87
Average B-factor	20.39
Macromolecules	18.10
Ligands	26.10
Solvent	33.42

*Statistics for the highest-resolution shell are shown in parentheses*.

### Protease activity assay

Six fluorogenic peptides, joined with an N-terminal Dabcyl group and C-terminal Edans group, were used as substrates and recombinant EV71 3C^pro^, 3C N69D, and 3C C147S protein were used as enzymes in the protease activity assays. Reactions were performed in a system containing 50 mM Tris-HCl (pH 7.0), 200 mM NaCl and 2 mM dithiothreitol (DTT) with a final concentration of 5.6 μM enzymes and substrates at different concentrations (3–45 μM) (Cui et al., 2011; Lu et al., 2011). After being mixed for 2 min, the relative hydrolysis rates were determined by monitoring fluorescence using a Cary Eclipse fluorescence spectrophotometer. The excitation wavelength was 340 nm, and emission was measured at 500 nm. Initial hydrolysis rates were recorded for each substrate concentration. Data were fitted to the Michaelis-Menten equation to calculate kinetic parameters K_m_, maximum rate of metabolism (V_max_), and K_m_/k_cat_ (Origin).

### ELISA

The concentrations of IFN-α in culture supernatants were measured by ELISA kits (R&D Systems). Briefly, RD cells were infected with H virus or L virus at MOI of 10 and cultured at 37°C for 6, 12, 24, and 36 h and then the culture supernatants were measured. Three experiments were performed for each time point.

## Results

### Construction of eight recombinant EV71 viruses

**Figure 1:**
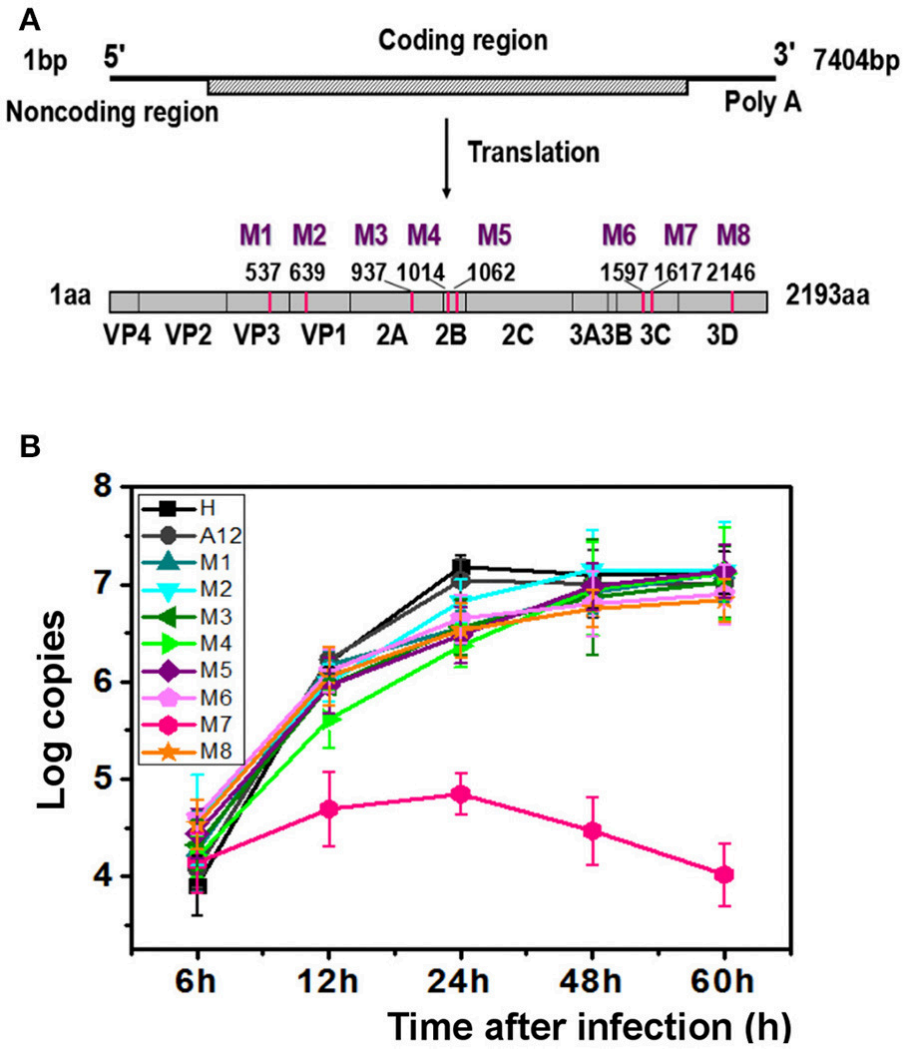
**M7 shows a different replication curve compared with wild type and other variants. (A)** Schematic illustration of the proteinic architecture of EV71 virus. The positions of residues that differ between the lethal strain and the non-lethal strain are marked as M1–M8. **(B)** Replication kinetics of wild-type EV71 and 8 recombinant viruses. The copies of EV71 genome replication in wild-type EV71 and 8 recombinant viruses were observed through RT-PCR. A12, a plasmid encoded complete cDNA of EV71, was employed to set up a standard curve. The proliferation curves demonstrated that the viral cDNA copies of M7 did not amplified as well as wild-type EV71 and other recombinant viruses.

Previously, we had isolated six EV71 strains from patients of the Jinan Infectious Diseases Hospital (Jinan, China) in 2008 and 2010 (Li et al., 2016). Animal experiments showed that these viruses exhibited different pathogenicity toward neonatal mice. Five of those viruses lead to the death of 1-day-old BALB/c mice and were designated the EV71 lethal-strain, and the one isolated from the mildly infected patient and conferred no symptoms to 1-day-old BALB/c mice was designated the non-lethal strain. Sequences of those viruses were published in Genebank: HQ825317, JQ074187, JQ074188, JQ074189, and JQ074190 for the lethal-strain and JF913464 for non-lethal strain. Interestingly, there are only eight differences in sequences of amino acids between the lethal-strains and the non-lethal strain (Table S1). To identify the genetic basis of the different pathogenicity of EV71 and the contributions of the amino acid substitutions to attenuation of virulence, eight single point recombinant virus were constructed based on A12 recombinant plasmid which include whole genome of a lethal-strain (GenBank no. HQ611148; Han et al., 2010; Cao et al., 2013; Li et al., 2015). Each variant included one amino acid replacement from the non-lethal strain, named M1 to M8 successively. The descriptions of those mutants are listed in Table S1, Figure 1A.

### Characterization of eight recombinant EV71 viruses

After transfection with same amount of RNAs, the typical cytopathic effect (CPE) appeared after 24 h in all genetically modified EV71 variants except M7. In parallel, the phenotype of CPE in M7-infected RD cells appeared after 2 days, weaker than the others (Figure S1). However, the expression of proteins by M1-M8 did not show significant differences when determined by immunoblotting at 24 h post infection, suggesting that differences in pathogenicity were not caused by differences in protein expression (Figure S2A). Additionally, indirect fluorescent-antibody (IFA) results showed that percentages of IFA-positive cells were obviously reduced in M7 but other mutants were more similar to WT (Figure S2B). The CPE was transmissible from the supernatant of the transfected cells for rescued EV71 virus variants to fresh cells expect for M7, which exhibited defective formation of new virus particles. We did not obtain the culturable virus of M7 despite multiple attempts. Replication kinetics of the rescued EV71 variants (M1, M2, M3, M4, M5, M6, M8) and WT virus (H, A12) were performed by quantitative RT-PCR using virus-infected cells with virus while M7 replication kinetics were determined with the transfected cell of RNA transcripts (Figure 1B). Results showed that mutant viruses grew as well as the WT virus but the intracellular RNA of M7 amplified weakly and tended to a decline after 24 h. The above data suggested that the single substitution in M7 may cause great changes in pathogenicity of EV71 virus. Bioinformatics analysis showed that the substitution of M7 was of the 69th residue of 3C protease, very close to the activity center of 3C protease based on the crystal structure of EV71 wild-type 3C^pro^ determined previously (Costenaro et al., 2011; Cui et al., 2011; Wang et al., 2011; Wu et al., 2013). Wang et al. reported that Asn69 of 3C^pro^ is associated with the hydrolytic activity of 3C protease (Wang et al., 2011).

### Crystal structure of 3C^pro^ N69D with rupintrivir

To determine the molecular mechanism underlying the above phenomenon, we used an x-ray crystallographic approach to determine the structure of the 3C N69D mutant. Perhaps due to conformational change, numerous screens for the crystallization of 3C N69D failed to give a hit but the 3C N69D-rupintrivir complex crystallized successfully. The structure was determined at 1.39 Ǻ resolution using a molecular replacement approach based on the previous 3C structure (PDB code: 3SJO) as searching model (Lu et al., 2011). The crystals belong to the *P*2_1_ space group, containing two 3C N69D-rupintrivir complexes per asymmetric unit. Data-collection and refinement statistics are presented in Table 1.

**Figure 2:**
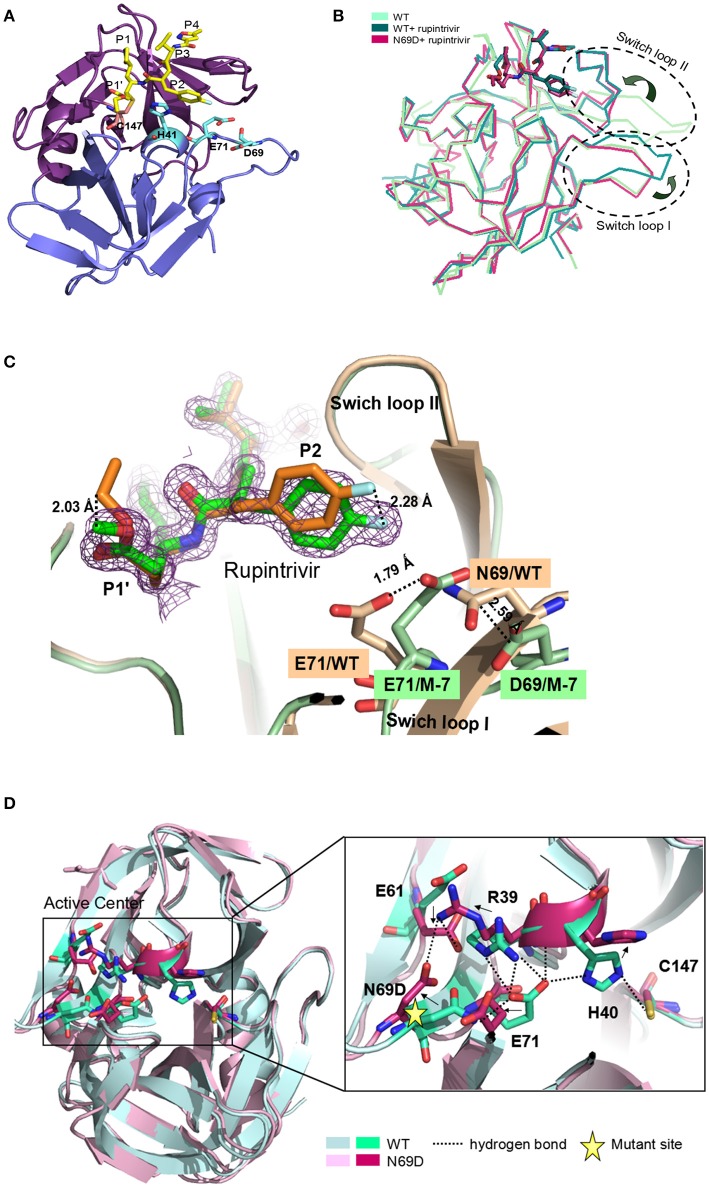
**Crystal structure of 3C^**pro**^ N69D with rupintrivir. (A)** Overall view of 3C^pro^ N69D-rupintrivir complex. Rupintrivir was shown as yellow sticks. **(B)** Binding of rupintrivir leads to different conformational changes for 3C and 3C N69D. The structure of WT 3C with or without rupintrivir (PDB no: 4GHT and 3OSY) are shown in blue and cyan, respectively. The 3C mutant N69D (PDB no: 5WQ2) is colored with hot pink. Two regions, responsible for substrate binding, are marked with dotted ovals and labeled as “Switch loop I” and “Switch loop II.” **(C)** Structural comparison of WT 3C-rupintrivir and 3C N69D-rupintrivir (PDB no: 4GHT and 5WQ2). WT 3C-rupintrivir and 3C N69D-rupintrivir are shown in yellow and green, respectively. Rupintrivir, Glu71, and Asp69/Asn69 are shown in the stick model. The 2.8-Å uplift of the P2 group of rupintrivir in 3C N69D relative to the position in WT 3C is indicated. The shifts of the positions of Glu71 and Asp69/Asn69 are marked and the distances between residues are annotated. **(D)** The N69D mutation leads to radical structural changes in the active center of 3C^pro^. Structural superposition of native 3C and 3C N69D was shown in cartoon model and residues in the active site are shown in stick model. Native 3C and 3C N69D are rendered in blue and pink, respectively. The hydrogen bond network of the active site of WT 3C is indicated with dashed lines. Residues involved in the hydrogen bond network of the active site are annotated. The shifts in the position of residues in active center are indicated with arrows.

Overall, the 3C^pro^ N69D folds into two domains similar to native 3C with well-defined electron density (Figure 2A). Structural alignment between the native 3C^pro^-rupintrivir complex (3SJO or 4GHT) and the 3C^pro^ N69D-rupintrivir complex yielded a root mean square deviation (RMSD) of 0.72–0.89 Å over 180 Cα-atom pairs as calculated by PDBeFold server. The structure of the 3C N69D-rupintrivir complex was superimposed with structures of the unliganded native 3C and 3C-rupintrivir complexes respectively (Figure 2B). As reported previously, there are two regions of 3C that are involved in rupintrivir and substrate binding, “switch loop I” and “switch loop II” (also known as β-ribbon; Birtley et al., 2005; Lee et al., 2009; Costenaro et al., 2011; Lu et al., 2011). Both switch loop I and switch loop II of the native 3C^pro^ demonstrated dramatic conformational changes after rupintrivir binding, converting from an “open” state to a “closed” state (Cui et al., 2011; Lu et al., 2011). However, unexpectedly, the switch loop II of 3C N69D-rupintrivir structure displays a “closed” state while the state of switch loop I remains “open” (Figure 2B). Structural analysis showed that the 69th residue located on one side of swich loop I, the N69D mutation leads to a series of comformation changes in the residues of the active center. As a result, the location of rupintrivir changed and switch loop I remains “open”in the structure of 3C N69D (Figures 2C,D).

Rupintrivir interacts with the active site of EV71 3C^pro^ via a network of hydrogen bonds and hydrophobic interactions. Unsurprisingly, the binding mode of rupintrivir in our structure is similar with those reported previously (Figure 2C; Costenaro et al., 2011; Cui et al., 2011; Lu et al., 2011). However, we observed significant discrepancies between the native 3C^pro^-rupintrivir complex and the 3C^pro^ N69D-rupintrivir complex at the P1′ and P2 sites of rupintrivir. First, the ester chain of P1′ group is exposed to the solvent in the native 3C structure, while it forms hydrogen bonds with the carbonyl oxygen of Gln22 in 3C N69D structure (Figure S3). As a result, the terminal atoms at P1′ in 3C N69D are in exactly the opposite direction compared to the ones in the native 3C structure. Second, according to the substitution of N69D, the P2 group makes a rotary movement of 2.28 Å relative to the position in native 3C. It is reported that the displacement of the P2 group of rupintrivir in EV71 3C and HRV 3C structure is only 1.6 Å despite a sequence identity of only 50% (Wu et al., 2013). The above results suggested that the single substitution of N69D may lead to obvious variation in the binding of 3C N69D to the inhibitor and substrate compared with native 3C^pro^.

### The overturned conformation in the active site of 3C^pro^ N69D

In the structures of most of active sites of picornaviral 3C^pro^, the catalytic triads His40, Glu71, and Cys147 adopt similar conformations that are stabilized via a series of hydrogen bonding interactions (Birtley et al., 2005; Lee et al., 2009; Zunszain et al., 2010; Costenaro et al., 2011; Cui et al., 2011; Lu et al., 2011). In particular, the side chain and main chain of Glu71 are hydrogen bonded by the surrounding residues in the structure of native EV71 3C-rupintrivir complex (dotted lines in Figure 2D). The glutamic acid side chain of Glu71 is immobilized by the hydrogen bonds from the backbone NH of His40, the Nδ2 atom of Asn69, and Nη2 & Nε atoms of Arg39. Wang et al suggested the firm conformation of catalytic center, especially residues Glu71, might be important for substrate recognition and catalytic activity (Wang et al., 2011). Unexpectedly, almost all the residues located in the active center of EV71 3C N69D exhibit dramatic changes when compared with the native 3C structure. Strikingly, expect for Cys147, the catalytic triads showed an overturned conformation never reported previously (Figure 2D). When Asn69 is replaced by Asp, the aspartic acid side chain of Asp69 uplifts and reverses 90°C forming a stable state via electrostatic interactions with the side chains of Arg39 and Glu61. As a result, the interaction between Glu71 and Asn69 is abrogated and there is a repellant force between two negative charge side chain residues. Furthermore, the hydrogen bonds donated by Arg39 are also disrupted because they are moved too far apart for interaction. Glu71 in 3C N69D structure shifts away from His 40 compared with native 3C, making the conformation of active center completely collapsed.

### Enzymatic activity of 3C^pro^ N69D decreased sharply both *in vitro* and *in vivo*

**Figure 3:**
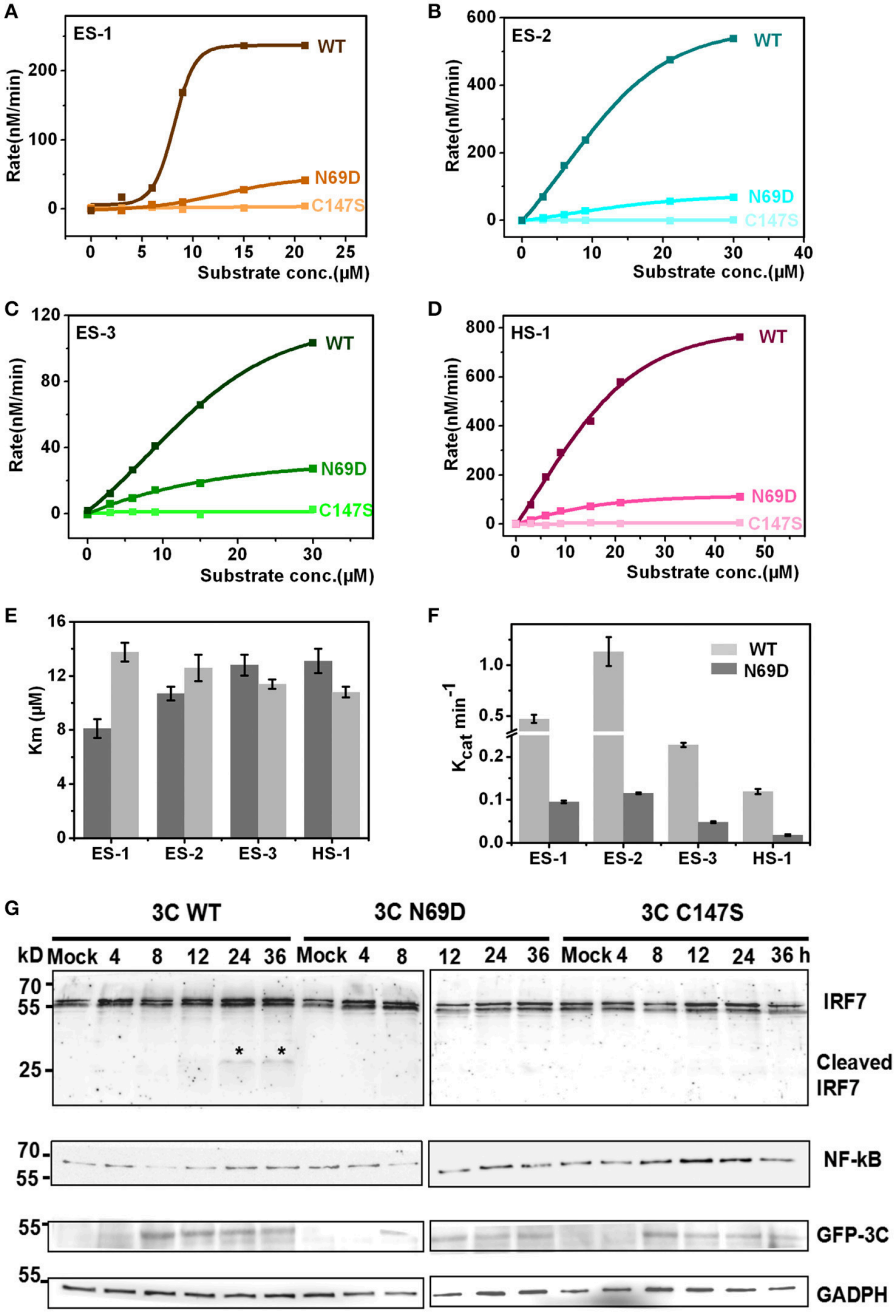
**Catalytic activities of native 3C and 3C N69D tested ***in vitro*** and ***in vivo***. (A)** The enzyme kinetics of native 3C and 3C N69D for the ES-1 peptide. The description of the fluorescence-labeled peptides is listed in Table S2. The concentration of the protease was 0.5 μM for all experiments. The initial velocities of the protease under a variety of different substrate concentrations were plotted against substrate concentrations to obtain the V_max_ and K_m_ values of the enzyme. The kinetic data were fitted with the Michaelis-Menten equation. **(B–D)** show the enzyme kinetics of WT 3C and 3C N69D to the ES-2 peptide, ES-3 peptide, and HS-1 peptide, respectively. Three independent measurements were performed. **(E,F)** Comparison of K_m_ and K_cat_ for the different substrates. K_m_ and K_cat_ (Vmax/[E]) were determined respectively. Error bars depict the standard deviation (S.D., *n* = 3). **(G)** Effect of WT 3C and 3C N69D on IRF7 and NF-κB in 293T cell. 293T cells were transfected with GFP or GFP-3C variants as indicated. The intracellular IRF7 and NF-κB was detected using immunoblotting by corresponding antibody at different times after transfection. GAPDH was included as an internal control.

The structure of 3C N69D reveals great changes in the active center indicating that 3C N69D may present different enzymatic activities and substrate specificities compared with native 3C^pro^. It has been reported that EV71 3C^pro^ is required not only for self-polyprotein processing during the virus replication but also participates in the disruption of the host immune system and metabolism by degrading host proteins during infection (Weng et al., 2009; Lei et al., 2010, 2011, 2013, 2014). To gain further understanding into the enzymatic properties of 3C^pro^ and 3C^pro^ N69D, we designed six fluorescent-labeled peptides, three based on the auto-processing sites of EV71 polyprotein and three corresponding to the reported host targets of EV71 3C^pro^ (Table S2). The substrates from EV71 are designated as ES-1, ES-2, and ES-3, and are based on the 10 residues between 2B–2C, 2C–3A, and 3B–3C junctions, respectively. HS-1, HS-2, and HS-3 are feasible substrates of 3C from the host targets CstF-64, IRF-7, and TRIF, respectively. All of those peptides include a Gln followed by a small residue (Gly or Ser) serving as the cleavage site of EV71 3C^pro^ and are joined with a N-terminal Dabcyl group and a C-terminal Edans group. When the peptide bond is cut off by the 3C protease, the fluorescence quenching pair Dabcyl-Edans is abrogated and fluorescence increase can be detected by fluorospectrophotometer. Using this method, we performed *in vitro* enzyme assays with native 3C^pro^, 3C^pro^ N69D, and 3C^pro^ C147S (a negative control was verified to be inactive) proteins purified from *E. coli*. Although we tried many conditions (data not shown), we still failed to detect the enzyme activity of 3C^pro^ to HS-2 and HS-3 because of the instability of HS-2 and HS-3 peptides. However, the reaction kinetics were successfully analyzed for 3C^pro^ and 3C^pro^ N69D against ES-1, ES-2, ES-3 and HS-1 (Figures 3A–D). 3C^pro^ and 3C^pro^ N69D present enzyme activity against peptides in different levels, and fluorescence increase was not observed in the system containing 3C^pro^ C147S. Three independent measurements were performed. The K_m_ values of 3C^pro^ and 3C^pro^ N69D against the three substrates (ES-2, ES-3, and HS-1) did not show significant difference, suggesting the substitution of Asn69 does not affect the affinity between 3C^pro^ N69D and those substrates despite the change of the charge in the active site. Remarkably, the K_m_ values of 3C^pro^ N69D against ES-1 containing a QS cleavage site showed an obvious increase indicating the N69D replacement may decrease the affinity between 3C^pro^ N69D and certain substrates (Figure 3E). More importantly, the K_cat_ values of 3C^pro^ N69D were reduced 5–10 times for all four substrates (Figure 3F). Most notably, the cleavage efficiency of 3C^pro^ N69D against 2B–2C junction decreased nearly ten times (Table S3). Our results show that 3C^pro^ N69D has a similar substrate binding affinity as wild-type 3C^pro^, but significantly lower enzymatic activity compared with native 3C^pro^.

To better understand the *in vivo* function, native 3C^pro^, 3C^pro^ N69D, and 3C^pro^ C147S were expressed in mammalian cells using a pFLAG-CMV2 vector which expressed target protein followed by green fluorescent protein. Previous studies have shown that wild type 3C^pro^ of EV71 reduces the expression of IRF7 by mediating IRF7 cleavage in infected cells (Lei et al., 2013). Therefore, we next detected the intracellular expression of IRF7 by immunoblotting. Interestingly, IRF7 was hydrolyzed in WT 3C^pro^-expressed cells but not in either 3C^pro^ N69D or 3C^pro^ C147S-expressing cells (Figure 3G). Previous studies showed that both EV71 2C and 3C protein targets components of the NF-κB pathway to inhibit the host's ability to defend against virus infection (Lei et al., 2014; Huang et al., 2015; Wang et al., 2015). We further examined real-time concentrations of NF-κB in native 3C^pro^, 3C^pro^ N69D, and 3C^pro^ C147S expressed cells by immunoblotting. The results showed that the concentration of NF-κB in 3C^pro^ C147S-expressed cells was elevated with the increased expression of 3C^pro^ C147S protein. In native 3C^pro^, the NF-κB level was decreased significantly at first and then slowly recovered to the initial concentration. Differently, the concentration of NF-κB in 3C^pro^ N69D-expressed cells was between the levels in native 3C^pro^ and 3C^pro^ C147S-expressed cells, suggesting that EV71 3C^pro^ could reduce the expression of NF-κB in a process closely related to its protease activity (Figure 3G). Together, these experimental results demonstrated that 3C^pro^ N69D has decreased enzyme activity and imperfect antagonism toward the host immune system compared to the WT 3C^pro^.

### N69D replacement of 3C^pro^ also occurs in other natural viruses

**Figure 4:**
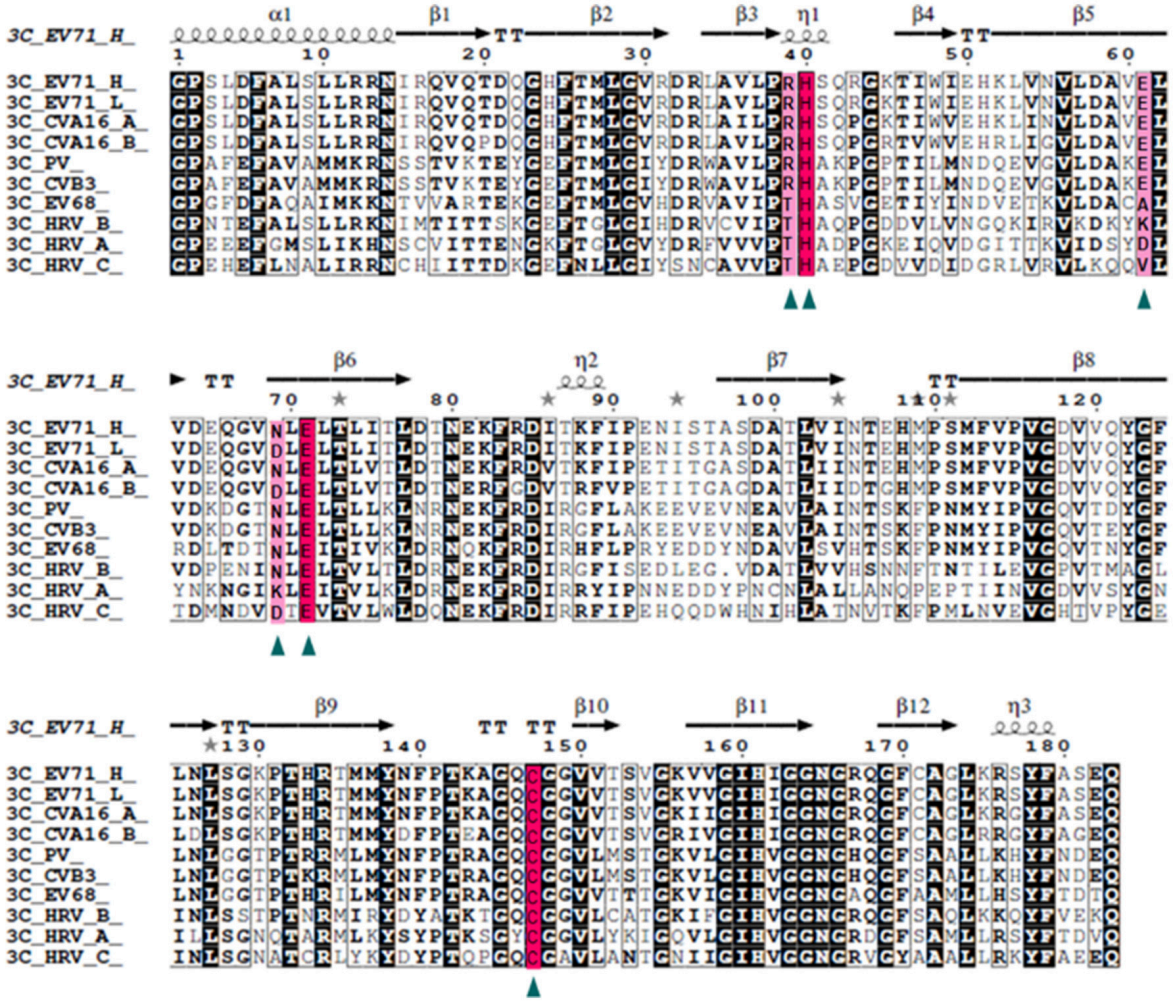
**Structure-based multiple sequences alignment of 3C homologs from EV71 and other picornaviruses**. The secondary structure and residue numbers are shown on the top. Conserved residues involved in catalytic activity are marked with triangles, The catalytic triad is highlighted with a hot pink background and others in light pink. Remarkably, the N69D mutation is present in some strains of EV71 and CAV16 and most stains of HRV_C.

Our data suggests that 3C^pro^ N69D acts as a subdued protease both *in vivo* and *in vitro*. To determine the frequency of this variation, we compared the EV71 sequence with five kinds of picornaviruses, CAV16, PV, CVB, EV68, and HRV, with 94, 72, 57, 54, and 50% identity, respectively (Figure 4). Structure-based sequence alignment showed that the catalytic triad is highly conserved in all selected viruses and some important residues (Arg39, Glu61, and Asp69) that participate in the hydrogen bonding network to stabilize the catalytic triad are less conserved. Most remarkably, the 3C^pro^ Asp69 also exists in other virus strains in addition to EV71. One strain of CAV16 isolated from Beijing in 2013 (GenBank code: KF193629) contains Asp rather than Asn in the 69th residue of 3C^pro^ sequence, suggesting that mutation in this site is not limited to the single case of EV71, but may be a secondary type of virus spreading between patients. More interestingly, we found that the Asp69 is present in HRV and surprisingly Asp69 was conserved in nearly all C-type HRV (HRV_C) closely related to asthma in children. No structure of HRV_C is available in PDB but we can speculate from our structure that the identity of the 69th reside may lead to great changes in substrate selection and catalytic activity of HRV_C. In most strains of A-type HRV, the 69th reside is positively charged Lys, in contrast to the negatively charged Asp studied here. Structural analysis reveals that Lys69 attracts Glu71 and Asp61 forming a stable conformation of the active site.

### Model for 3C N69D-mediated attenuation of EV71 virulence

**Figure 5:**
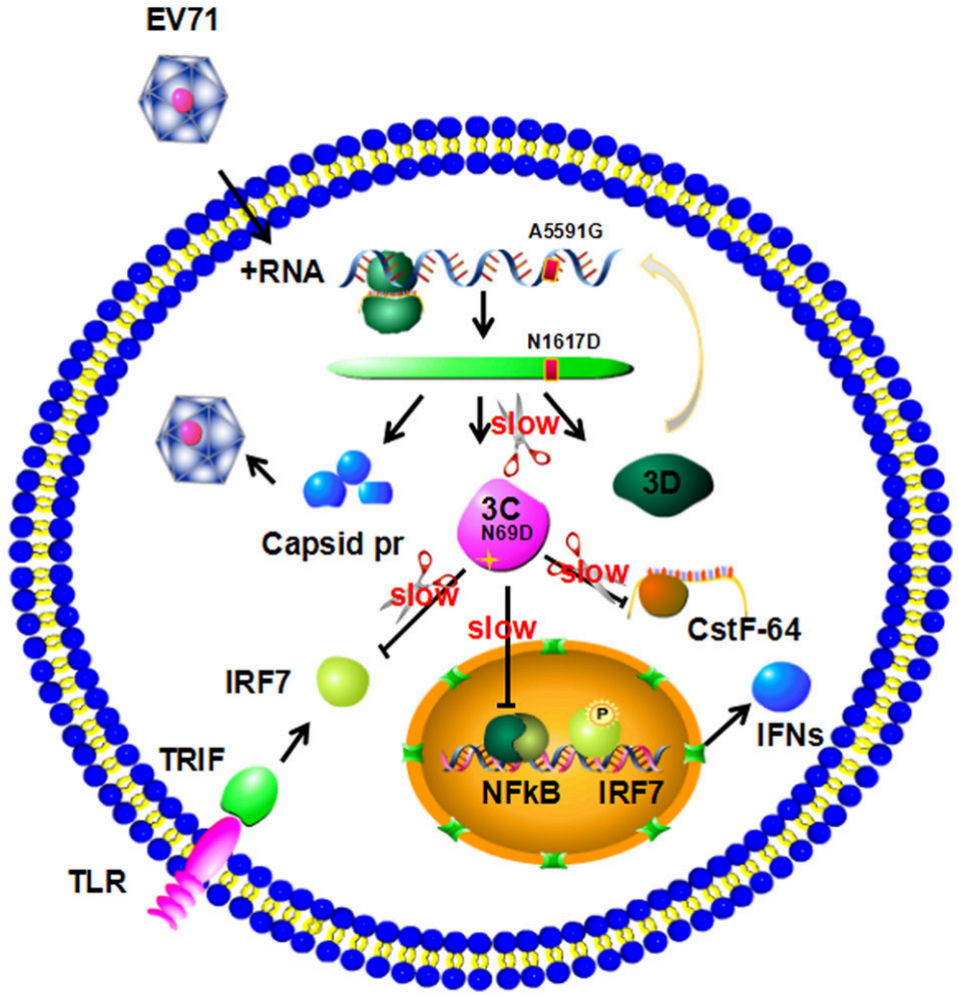
**Model for 3C N69D-mediated attenuation of EV71 virulence**. The N69D substitution of 3C^pro^ decreases viral reproductivity due to a slower processing rate of viral precursor polypeptides. The N69D mutation can also affect the ability of 3C^pro^ to promote the shutoff of host cell metabolism by hydrolysis of CstF-64 and other factors. Most importantly, this substitution weakens the inhibition of host immune system mediated by protease activity of 3C by hydrolysis of IRF-7 and inhibition of NF-κB pathway. Taken together, the 3C N69D mutation was responsible for the attenuation of EV71 virulence.

The above data suggest that the N69D substitution may explain the attenuation of virulence of the non-lethal strain. First, the N69D substitution of 3C^pro^ will result in a subdued viral reproductivity due to a slower speed to process viral precursor polypeptides. Previous studies have demonstrated that the replication ability of the lethal strains were significantly higher than the non-lethal strain (Li et al., 2016). Second, our result showed that the hydrolytic activity of 3C N69D to host target CstF-64, the polyadenylation protein involved in the 3′ end cleavage and polyadenylation of pre-mRNAs, was 15% of native 3C. As a result, the function of 3C^pro^ involved in the shutoff of host cell metabolism might also be affected by the N69D substitution. Then the inhibition of host cellular transcription by 3C^pro^ could be decreased. More importantly, the inhibition of host immune system mediated by protease activity of 3C will be crippled as well (Figure 5). To test our hypothesis, we measured the intracellular production of IFN-α/ IFN-β after infection of one lethal strain (H) and one non-lethal strain (L) using ELISA kit. Consistent with our expectations, the concentration of IFN-α in the non-lethal strain-infected cells was much higher than that of lethal strain-infected cells in 6, 12, and 24 hpi then tend to be similar in 36 hpi (Figure S4). However, IFN-β was not detected in either the lethal strain or the non-lethal strain-infected cells.

## Discussion

In China, hundreds of children are killed by severe hand, foot and mouth disease every year and the culprit of this severe HFMD is the EV71 virus (McMinn, 2002; Yang et al., 2009). However, unlike the poliovirus, no effective drug or vaccine against EV71 is available, which makes it a serious threat to the health of children and infants in China and the rest of the world (Lum et al., 1998; Huang et al., 1999). This virus belongs to the picornaviridae family and consists of a positive single-stranded RNA that exhibits frequent variation (Lum et al., 1998; Ho, 2000). Previous research on the relationship between viral genetic sequences and virulence mainly focused on two proteins: VP1 and 3D (Parks et al., 1989; Meng and Kwang, 2014; Zhang et al., 2014; Bhakat, 2015; Yuan et al., 2015). Some reports showed that a single mutation of VP1 or 3D would lead to great changes in the severity of symptoms. However, little is known about the molecular mechanisms underlying these phenotypes. Furthermore, both the patient's immune system and viral genetic sequences contribute to the severity of symptoms so that in other words, the same virus will cause different symptoms in different persons (Huang et al., 1999; Weng et al., 2010). Therefore, mice with the same genetic background are developed as appropriate models to research the relationship between viral genetic sequences and patient symptoms.

Our group developed neonatal mice models of the EV71 infection and focused on the mechanisms of mild and severe symptoms of HFMD, which were caused by slight differences in genomic sequence. Six EV71 strains that were isolated from patients of Jinan, in 2008 and 2010, exhibited different pathogenicity toward neonatal mice (Li et al., 2016). Only eight differences were found in the amino acid between lethal-strains and non-lethal strains. To find out the mutation(s) responsible for the attenuated virulence, eight single point recombinant virus variants were constructed based on a lethal-strain. The one that included a substitution in the 69th residue of 3C protease exhibited different phenotypes compared with the wild type EV71 and the other reassortant viruses. To better understand this phenomenon, the structure and function of 3C N69D were explored in detail. To do this, we first crystallized and solved the structure of 3C N69D-rupintrivir complex. We found that the N69D mutation disrupted one of the substrate binding switches and presented an altered active site conformation. Second, we discovered that, consistent with the apparent structural disruption, the catalytic activity of 3C N69D to substrates became only 1/5–1/10 of the normal 3C protein, which suggests the 69th of the 3C protease was a significant regulatory site of 3C activity. Further, the *in vivo* data showed that the inhibitory ability of 3C N69D to the host targets, IRF7 or NF-κB, was not as effective as the wild-type 3C protein. All above data suggests the replication speed of the virus, which contains the 3C N69D mutation, will be slower. Further to this, the data proposes the inhibitory function of EV71 to the host's immune system, which occurs by decomposing central immune molecules, will also be crippled thus significantly weakening the pathogenicity of EV71.

One of the deficiencies, however, in this study was that we did not obtain the culturable virus of M7, despite multiple attempts throughout the course of our research. The reasons are as follows. First, by using a normal concentration of RNAs, the activity of 3C N69D may be too low to produce enough viral proteins to form new virus particles within a short time. Second, if we use a high concentration of RNAs, a large number of viral proteins will be produced and the typical cytopathic effect would appear too fast, which would restrict the formation of new virus particles.

3C protease is one of the most important proteins involved in the virus-host interaction and serves as a potent target for designing anti-EV71 agents (Parks et al., 1989; Reich et al., 2000; Li et al., 2002; Kuyumcu-Martinez et al., 2004b; Kundu et al., 2005; Kuo et al., 2008). Our findings broaden our understanding to the catalytic mechanism of 3C protease and provide new insights into the structure and function of a subdued 3C protease mutant, which exists in a wide variety of viruses. It is one of the most popular research topics: to identify novel cellular proteins as host substrates of 3C proteases. Our research showed that the non-lethal virus we isolated from a mild patient, which includes the 3C N69D mutant, exhibited obvious differences in its ability to hydrolyze substrates. So more host substrates of 3C protease will be identified according to the differences in concentration of proteins of lethal virus and non-lethal virus-infected cells. Moreover, the N69D replacement of 3C^pro^ is identified in a variety of natural viruses, so detection of this variation might contribute to the prediction of clinical presentation. In addition to this, the attenuated non-lethal EV71 variants, with robust replication profiles, would contribute to the development of both live attenuated and inactivated EV71 vaccines. However, despite what our research has accomplished, further data will be needed to show the significance and universality of N69D replacement of 3C protease as demonstrated in the present study.

## Accession codes

Atomic coordinate and structure factor of 3C N69D has been deposited in the Protein Data Bank (http://www.rcsb.org) under accession number 5WQ2.

## Author contributions

BL and HM designed the study; YY and YZ performed functional experiments; BL, ZY and LG performed structural experiments; PL, NS and WL analyzed data; YL purified 3C protein; BL wrote the manuscript.

### Conflict of interest statement

The authors declare that the research was conducted in the absence of any commercial or financial relationships that could be construed as a potential conflict of interest.

## Supplementary material

Figure S1 **The typical cytopathic effect of eight recombinant EV71 viruses**. The typical cytopathic effect (CPE) of modified EV71 variants transfected with same amount of RNAs was shown in this picture. **(A)** Uninfected control; **(B)** Cells infected by M1 RNA for 24 h (A12 and other 6 mutations except M7 showed same phenotype, data not shown); **(C)** Cells infected by M7 RNA for 48 h.

Figure S2 **Immunoblotting and Indirect fluorescent-antibody results of WT EV71 and recombinant EV71 viruses. (A)** Immunoblotting results of cells transfected with RNAs of WT or recombinants detected by 3C and 3D antibody. GADPH was detected as a control. Samples were prepared at 24 h after infection. H one of lethal strains (GenBank accession no. HQ825317); A12: recombinant EV71 virus of a lethal strain (GenBank accession no. HQ611148); M1–M8 recombinant EV71 virus with single residue replacement (Table S1). **(B)** Indirect fluorescent-antibody results detected by mouse polyclonal antibody to EV71. Percentages of IFA-positive cells were obviously reduced in M7 but other mutants were more similar to WT.

Figure S3 **N69D mutant leads to different orientation in P1′ group of rupintrivir**. WT 3C-rupintrivir and 3C N69D -rupintrivir are shown in yellow and green, respectively. Rupintrivir, Gln 22, His 40, Glu71, and Asp69/Asn69 are shown in the stick model. the ester chain of P1′ group is exposed to the solvent in the native 3C structure, while it forms hydrogen bonds with the carbonyl oxygen of Gln22 in 3C N69D structure.

Figure S4 **The intracellular production of IFN-α in lethal strain and non-lethal strain infected cell**. The concentrations of IFN-α in culture supernatants of H strain (one of lethal strains, GenBank accession no. HQ825317) or L strain (the non-lethal strain, GenBank accession no. JF913464) infected cells were analyzed by ELISA kits.

Table S1 **Sequence alignment results of lethal strains and non-lethal strain**.

Table S2 **Information of peptides used in protease activity assay**.

Table S3 **Cleavage efficiencies of EV71 3C WT and N69D mutant**.
