# Supplementary figures and images for: A Novel Enterovirus 71 (EV71) Virulence Determinant: The 69th Residue of 3C Protease Modulates Pathogenicity
Source: Front Cell Infect Microbiol. 2017 Feb 3;7:26. doi: 10.3389/fcimb.2017.00026 (PMC5290453; doi:10.3389/fcimb.2017.00026)

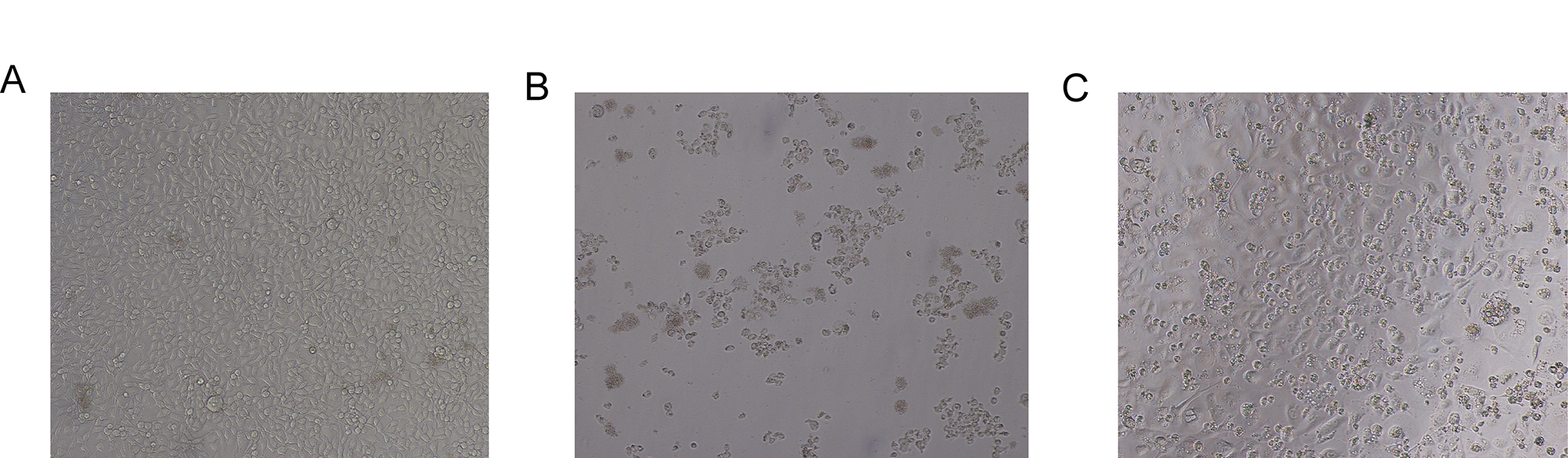

Supplement: Figure S1 — The typical cytopathic effect of eight recombinant EV71 viruses. The typical cytopathic effect (CPE) of modified EV71 variants transfected with same amount of RNAs was shown in this picture. (A) Uninfected control; (B) Cells infected by M1 RNA for 24 h (A12 and other 6 mutations except M7 showed same phenotype, data not shown); (C) Cells infected by M7 RNA for 48 h. [file Image1.TIF]

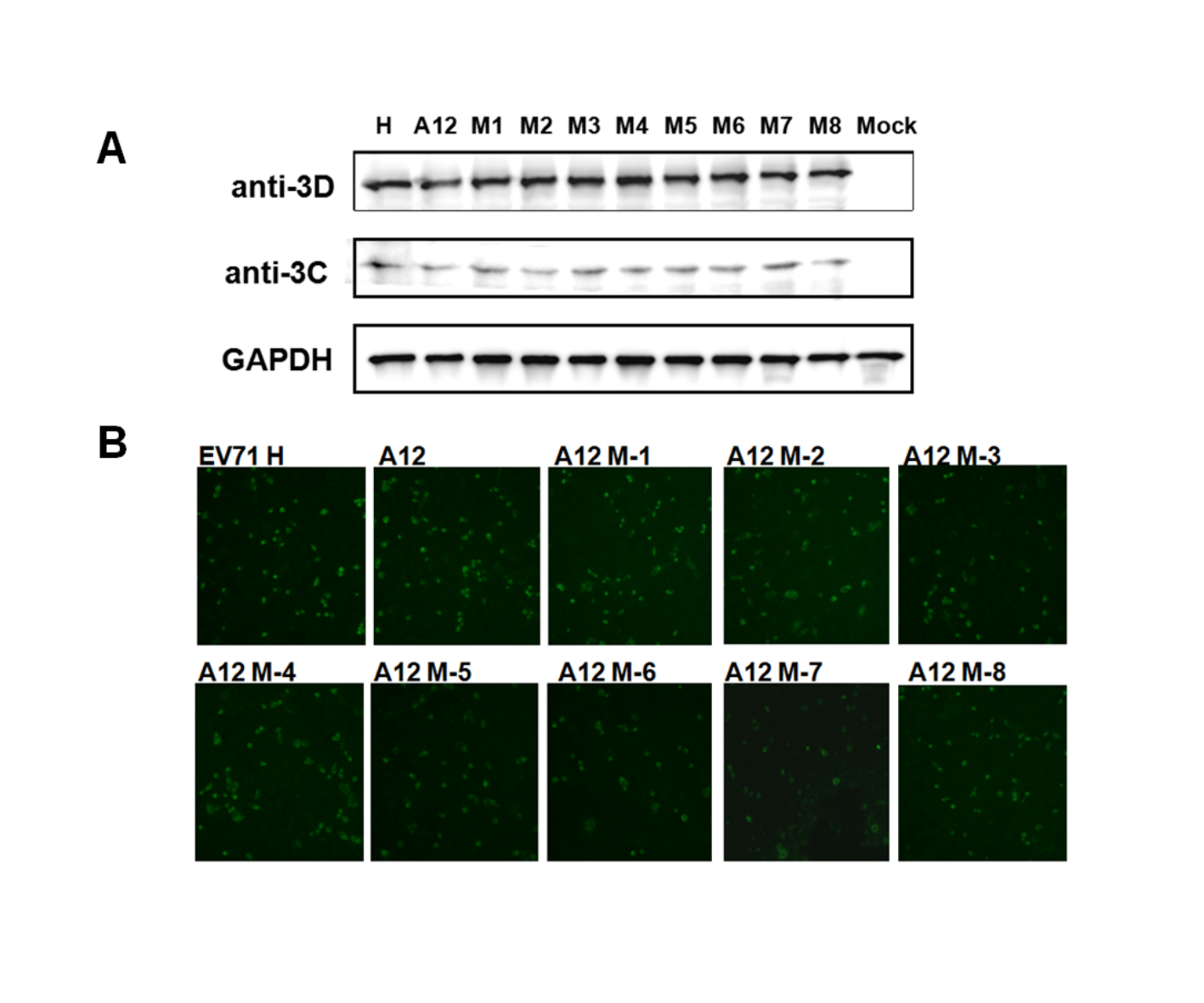

Supplement: Figure S2 — Immunoblotting and Indirect fluorescent-antibody results of WT EV71 and recombinant EV71 viruses. (A) Immunoblotting results of cells transfected with RNAs of WT or recombinants detected by 3C and 3D antibody. GADPH was detected as a control. Samples were prepared at 24 h after infection. H one of lethal strains (GenBank accession no. HQ825317); A12: recombinant EV71 virus of a lethal strain (GenBank accession no. HQ611148); M1–M8 recombinant EV71 virus with single residue replacement (Table S1). (B) Indirect fluorescent-antibody results detected by mouse polyclonal antibody to EV71. Percentages of IFA-positive cells were obviously reduced in M7 but other mutants were more similar to WT. [file Image2.TIF]

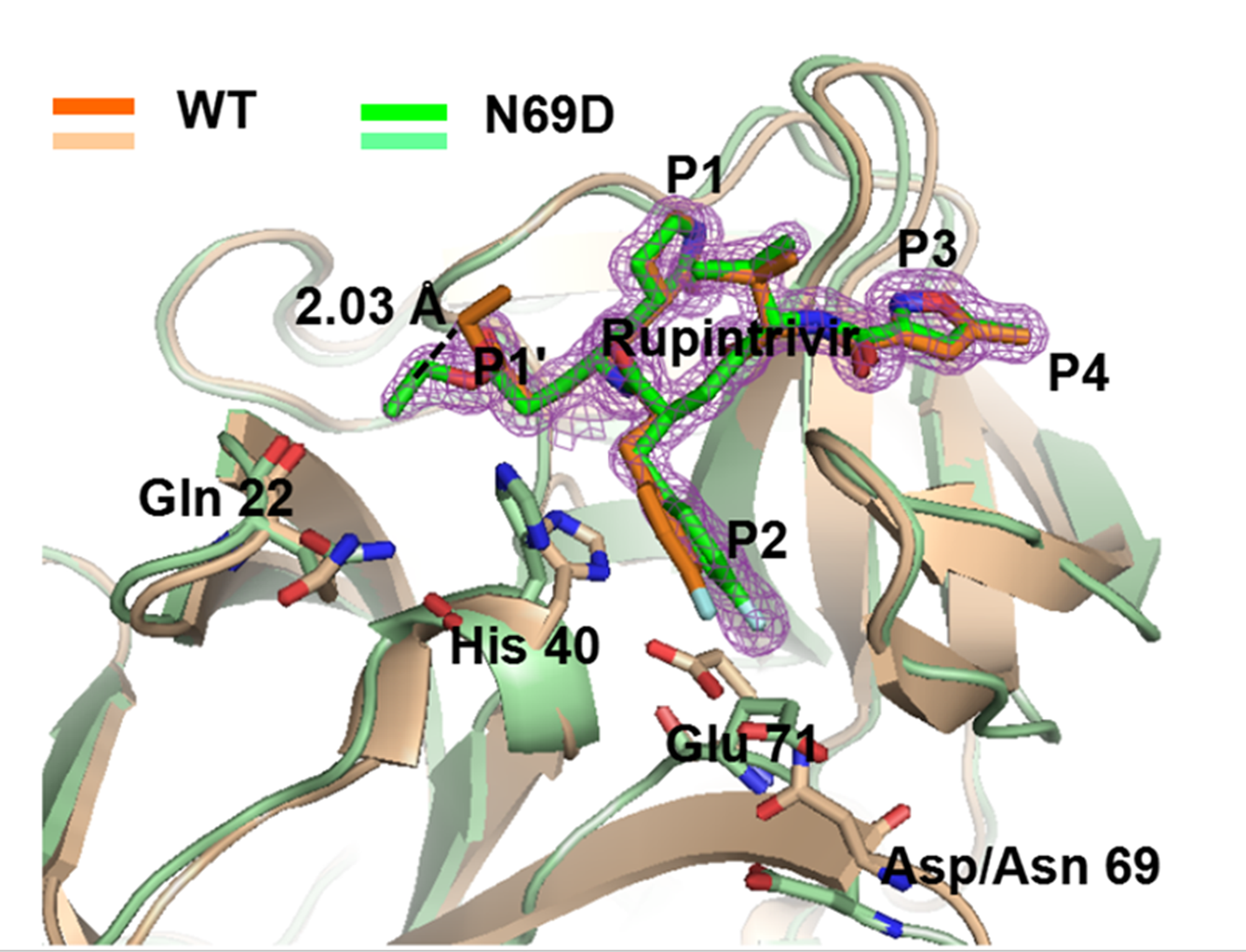

Supplement: Figure S3 — N69D mutant leads to different orientation in P1′ group of rupintrivir. WT 3C-rupintrivir and 3C N69D -rupintrivir are shown in yellow and green, respectively. Rupintrivir, Gln 22, His 40, Glu71, and Asp69/Asn69 are shown in the stick model. the ester chain of P1′ group is exposed to the solvent in the native 3C structure, while it forms hydrogen bonds with the carbonyl oxygen of Gln22 in 3C N69D structure. [file Image3.TIF]

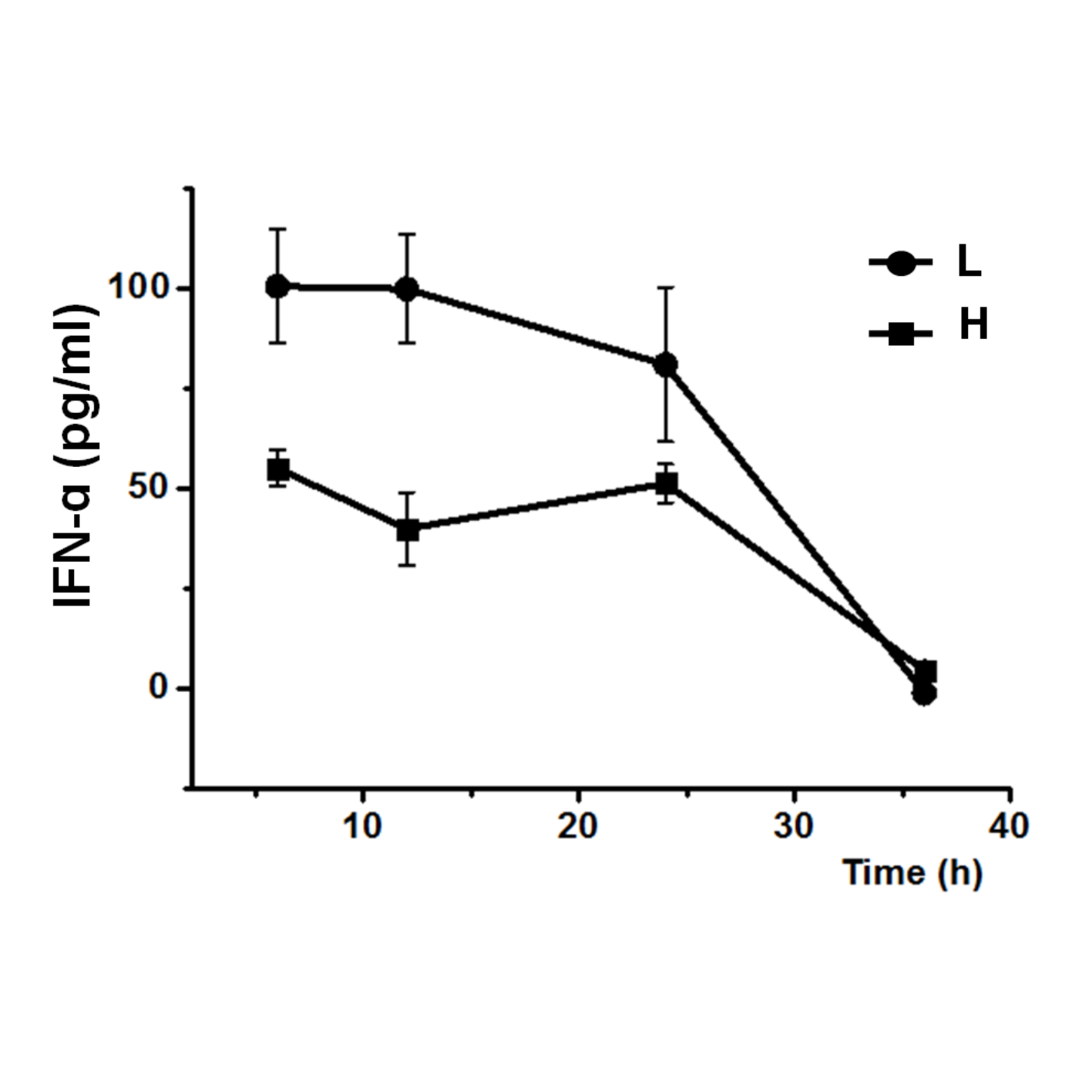

Supplement: Figure S4 — The intracellular production of IFN-α in lethal strain and non-lethal strain infected cell. The concentrations of IFN-α in culture supernatants of H strain (one of lethal strains, GenBank accession no. HQ825317) or L strain (the non-lethal strain, GenBank accession no. JF913464) infected cells were analyzed by ELISA kits. [file Image4.TIF]
