# Supplementary material for: A Novel Enterovirus 71 (EV71) Virulence Determinant: The 69th Residue of 3C Protease Modulates Pathogenicity
Source: Front Cell Infect Microbiol. 2017 Feb 3;7:26. doi: 10.3389/fcimb.2017.00026 (PMC5290453; doi:10.3389/fcimb.2017.00026)
Supplement: Table S1 — Sequence alignment results of lethal strains and non-lethal strain. [file Table1.DOC]

TABLE S1 Sequence alignment results of lethal strains and non-lethal strain

| Number | Related Protein | Mutative Sites  (lethal strain→  non-lethal strain) | Nucleotide Sites  (lethal strain→  non-lethal strain) |
| --- | --- | --- | --- |
| M-1 | VP3 | 537 (I→V) | 2351 (A→G) |
| M-2 | VP1 | 639 (S→G) | 2657 (A→G) |
| M-3 | 2A | 937 (S→G) | 3551 (A→G) |
| M-4 | 2B | 1014 (V→A) | 3783 (T→C) |
| M-5 | 2B | 1062 (I→T) | 3927 (T→C) |
| M-6 | 3C | 1597 (V→I) | 5531 (G→A) |
| M-7 | 3C | 1617 (N→D) | 5591 (A→G) |
| M-8 | 3D | 2146 (V→A) | 7179 (T→C) |
