# Supplementary material for: A Novel Enterovirus 71 (EV71) Virulence Determinant: The 69th Residue of 3C Protease Modulates Pathogenicity
Source: Front Cell Infect Microbiol. 2017 Feb 3;7:26. doi: 10.3389/fcimb.2017.00026 (PMC5290453; doi:10.3389/fcimb.2017.00026)
Supplement: Table S2 — Information of peptides used in protease activity assay. [file Table2.DOC]

**TABLE S2 Information of peptides used in protease activity assay**

| Peptides | Polyprotein junction/  Host substrate | Function of substrate | Sequence  (N→C) |
| --- | --- | --- | --- |
| ES-1 | 2B-2C | the autoprocessing sites of EV71 | LAQKQSASWL |
| ES-2 | 2C-3A |  | EALFQGPPKF |
| ES-3 | 3B-3C |  | TATVQGPSLD |
| HS-1 | CstF-64  **C**leavage **st**imulation **f**actor **64** kDa subunit | [polyadenylation protein](http://www.baidu.com/link?url=-vDi9lAkzkwamA5foE7fKdRyWcBSpWuBfLjr1peCCV_Ta5abGjRHcPYnzgy3jXzm-ZovJ5qgSwBbeZA9iLB5Ia) involved in the 3' end cleavage and polyadenylation of pre-mRNAs | QASMQ_251_GGVPA |
| HS-2 | IRF-7  **I**nterferon **r**egulatory **f**actor **7** | transcriptional activation of virus-inducible cellular genes including the type I interferon genes | QAVQQ_189_SCLAD |
| HS-3 | TRIF  TIR domain  containing adapter inducing interferon β | an adapter in responding to activation of [toll-like receptors](http://en.wikipedia.org/wiki/Toll-like_receptors) (TLRs) | SAGPQ_312_SLPLP |
