# Supplementary material for: A Novel Enterovirus 71 (EV71) Virulence Determinant: The 69th Residue of 3C Protease Modulates Pathogenicity
Source: Front Cell Infect Microbiol. 2017 Feb 3;7:26. doi: 10.3389/fcimb.2017.00026 (PMC5290453; doi:10.3389/fcimb.2017.00026)
Supplement: Table S3 — Cleavage efficiencies of EV71 3C WT and N69D mutant. [file Table3.DOC]

| **No.** | **Affinity to substrate**  **(Km μM)** | | **Cleavage efficiency**  **(K_cat_ min^-1^)** | | |
| --- | --- | --- | --- | --- | --- |
|  | **3C WT** | **3C N69D** | **3C WT** | **3C N69D** | **WT/N69D** |
| **ES-1** | **8.1±0.7** | **13.8±0.69** | **0.473 ± 0.04** | **0.095 ± 0.003** | **4.98** |
| **ES-2** | **10.7±0.5** | **12.6±0.98** | **1.132 ± 0.14** | **0.115 ± 0.002** | **9.84** |
| **ES-3** | **12.8±0.78** | **11.4±0.35** | **0.228 ± 0.005** | **0.048 ± 0.002** | **4.75** |
| **HS-1** | **13.1±0.9** | **10.8±0.4** | **0.119 ± 0.006** | **0.018 ± 0.002** | **6.80** |

**TABLE S3 Cleavage efficiencies of EV71 3C WT and N69D mutant**
